# Chaotic dynamics in X-ray free-electron lasers with an optical undulator

**DOI:** 10.1038/s41598-024-51891-1

**Published:** 2024-01-16

**Authors:** E. Abbasi, S. Jafari

**Affiliations:** Department of Physics, Faculty of Science, University of Guilan, Rasht, 41335-1914 Iran

**Keywords:** Applied physics, Plasma physics, Statistical physics, thermodynamics and nonlinear dynamics

## Abstract

In this work, the chaotic motions of relativistic electrons in X-ray free-electron lasers are investigated using an optical undulator in the presence of a magnetized ion-channel background. To miniaturize X-ray light sources, the optical undulator is a promising concept. The optical undulator provides higher optical gain than conventional magnetostatic undulators due to its micrometer wavelength. In addition, it reduces the required electron beam energy from several GeV to the multi-MeV range to produce X-ray pulses. The interaction of an optical undulator with an intense relativistic electron beam is a highly non-linear phenomenon that can lead to chaotic dynamics. At synchrotron radiation sources, the possibility of chaos control for X-ray FELs can be critical for certain classes of experimental studies. The equations of motion for a relativistic electron propagating through the optical undulator in the presence of a magnetized ion-channel can be derived from the Hamiltonian of the interaction region. Simulation results revealed that the intensity of the perturbation route from orderly behavior to chaos depends on the beam density, axial magnetic field strength, ion-channel density parameter, and pump laser undulator. Specific values of parameters were obtained for the transition from regular to chaotic paths. Bifurcation diagrams of the system were plotted to demonstrate the origin of chaos at a critical point, and Poincaré maps were created to distinguish between chaotic and orderly motions of electrons. The proposed new scheme can help to improve X-ray FELs, which have potential usages in basic sciences, medicine, and industry.

## Introduction

As a cutting-edge research instrument, X-ray free-electron lasers (XFELs) can generate fully coherent radiation with high power and short-pulse length^1–3^. The XFEL's unique properties, such as wavelengths well into the hard X-ray region and high peak brilliance, facilitate novel investigations involving ultrafast single-particle imaging, non-linear X-ray spectroscopy, chemical transformations, and structural and functional biology^4–6^. In recent years, XFELs have been designed to generate radiation starting from the shot noise of a relativistic electron beam, the so-called self-amplified spontaneous emission (SASE) mechanism^7,8^. However, the main drawbacks of SASE-XFELs are relatively poor longitudinal coherence and a long conventional magnetostatic undulator is required^9,10^. Moreover, to realize SASE-XFELs at angstrom wavelengths, extremely high brilliance e-beam, and beam energies of order GeV are required^11,12^. Hence, several alternative concepts are under consideration for the feasibility of XFELs, and in this regard, optical undulators have attracted much attention^13–17^.

The optical undulator due to its very short undulator wavelength (in the micrometer range) provides both a shorter output wavelength (in the hard X-ray region) and a higher optical gain relative to the common magnetostatic undulators^13,18,19^. Besides, it reduces the required e-beam energy from several GeV to the multi-MeV range to generate X-ray pulses^20,21^. With remarkable progress in producing terawatt laser pulses and optical fibers, the optical laser undulator can prepare an impressive magnetic field of the order of kilo-Tesla, which can yield an intense effective undulator strength^22,23^. In a laser-driven XFEL, an intense electron bunch propagates through a laser undulator and generates a high-power X-ray pulse (of the order of several tens of GW) with a narrow bandwidth (< 0.01%)^24–26^. In contrast to Compton scattering sources, which have a short interaction region, the laser undulators (generating X-rays through Thomson backscattering) have a long interaction region, and hence require a focusing arrangement to transport the electron bunch through their comparably small aperture^27,28^. Since the laser beam size varies with travel distance, accordingly, the local undulator strength varies during the head-on interaction of an electron bunch and a laser beam. In fact, from the experimental point of view, it may be difficult to maintain resonance conditions in laser undulators, since it is troublesome to hold the focus of the pump wave over a significant distance to achieve amplification^29^. This case is fatal for a high-gain FEL, which needs to maintain the required resonance condition. To suppress this issue, it needs to prevent laser beam size variations so that the local power of the laser undulator does not change during resonant interaction. Moreover, another serious obstacle to intense e-beam transport in XFELs is the transverse e-beam breakup instability^30^. These drawbacks related to laser beam focusing and intense electron beam transmission can be suppressed by employing a magnetized ion-channel (or originally a magnetized plasma background)^31–34^. The ion-channel generated by the plasma medium can also withstand high fields due to being in the ionized state and therefore overcome the restriction of material breakdown^35,36^. Introducing a magnetized ion-channel into the interaction region can confine the e-beam and maintain the focus of the pump wave over a significant distance^37,38^. The introduction of ion-channel in laser-driven-XFELs helps to reduce the e-beam energy requirement and enhances the power of X-ray pulses^38^.

In a high-power FEL operating at high electron beam currents, the stability of the electron beam is particularly important to achieve high gain and efficiency. Under this condition, the electron motion is altered by the self-electric and self-magnetic fields induced by the charge density and current density of the e-beam. The interaction of a laser beam with an intense relativistic electron beam is a highly non-linear phenomenon and can lead to chaotic dynamics^39,40^. In the case of synchrotron radiation sources, the possibility of controlling chaos for FELs can be crucial for specific classes of experimental studies^40,41^. In this work, we simulate chaotic e-beam dynamics in the interaction of an antiparallel propagating linearly polarized laser pulse (as an optical undulator) with an intense relativistic e-beam in the presence of a magnetized ion-channel background. Poincaré maps are also generated by numerically integrating the Hamiltonian-derived equations of motion in the interaction region to determine between regular and chaotic behavior at resonance. At X-ray wavelengths, linearly polarized undulator has been employed for multiple reasons, including the desire for high magnetic fields at short undulator periods to maximize FEL gain and lower construction cost. A lot of trouble to extract a circularly polarized laser as an undulator, while a linearly polarized laser can be achieved using a conventional quantum laser. It is also believed that linearly polarized undulators have lower error fields than those generally found in variable polarization undulators^42^. The proposed novel scheme can help to improve X-ray FELs, which have potential usages in fundamental sciences, medicine, and industry.

## Results

**Figure 1:**
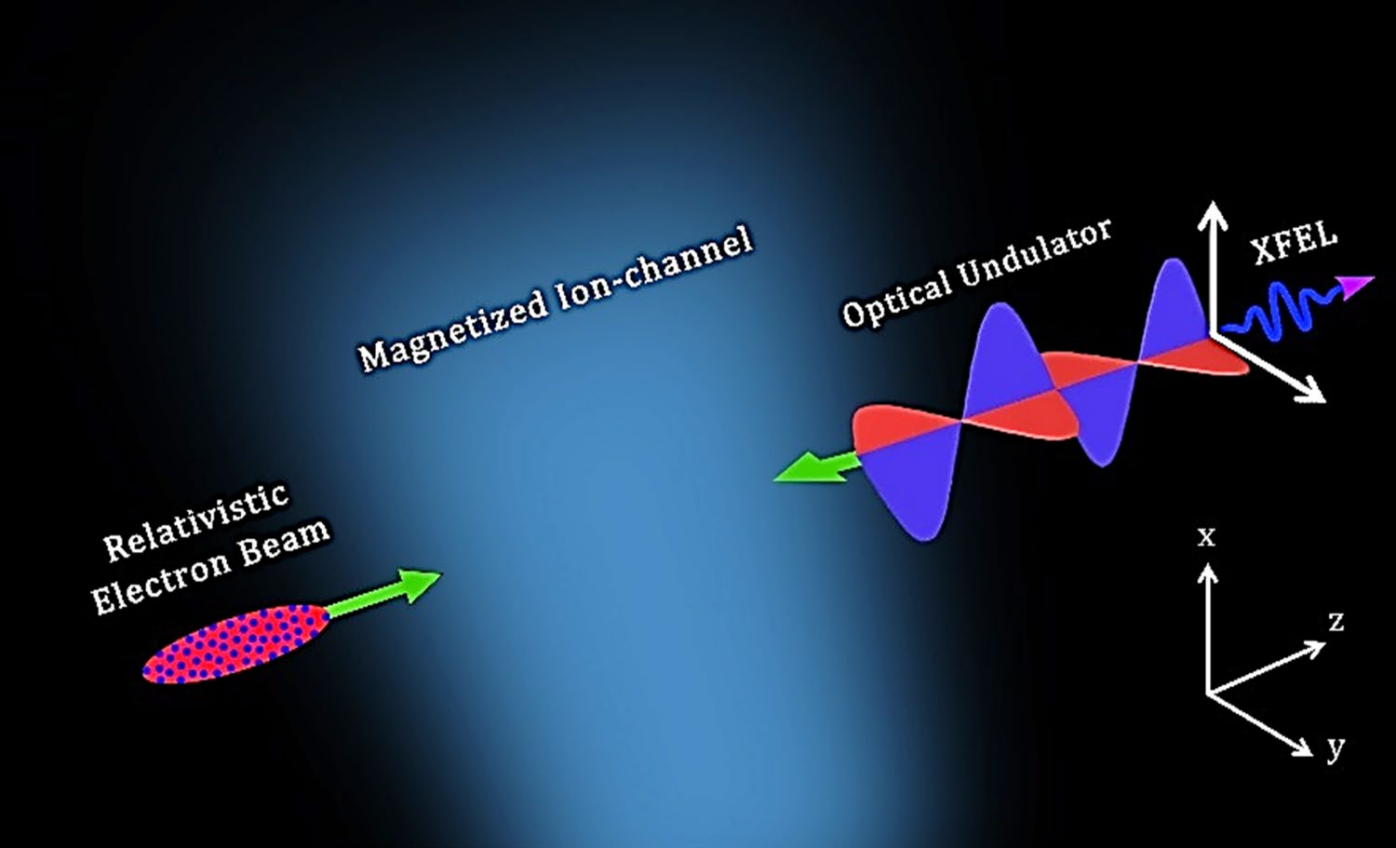
An X-ray FEL scheme based on an optical undulator in the presence of a magnetized ion-channel background.

The schematic illustration of the X-ray FEL based on an optical undulator with a magnetized ion-channel background has been presented in Fig. 1. As a relativistic e-beam moves axially, the transverse orbit of the electron becomes unstable due to the beam self-fields effect^43^. Abbasi et al*.*^15^ investigated the interaction of an intense electron beam with a laser pulse (as an optical undulator) using an external magnetic field as a beam guide. It was shown that self-fields significantly affect the FEL gain in the resonant region. This effect is related to the chaos phenomenon and will be evaluated in this study. The existence of chaotic dynamics in XFELs reduces the gain and efficiency. Therefore, finding ways to suppress the beam instability and create stable trajectories is one of the important challenges in XFELs. In this work, we evaluate the influences of the magnetized ion-channel background on the control of chaotic pathways. The results of chaos simulation based on an optical undulator are given in this section.

For evaluating chaos in XFELs, the first step is to examine the bifurcation diagrams. The bifurcation and the chaotic transition have become an important subject in XFELs, and surveying them is useful for specifying optimal operating regimes. Bifurcation diagram is a classical tool to investigate the dynamics of non-linear system. It is a visual representation of the behavior of a system as a parameter changes. Generally, bifurcation diagrams indicate a complex sequence of bifurcations in which the path to developed chaos is interrupted by windows of periodic or quasi-periodic behaviors. Another method to investigate the non-linear dynamics in XFELs is generating the Poincaré maps. A Poincaré map can be interpreted as a discrete dynamical system with a state space that is one dimension smaller than the original continuous dynamical system. We provide a detailed explanation of the theory and fundamental equations governing the bifurcation diagrams and the Poincaré maps in the non-linear regime of electron beam-undulator interaction in the “Methods” section. To obtain bifurcation and Poincaré cross-section diagrams, we employ the fourth-order Runge–Kutta method to integrate the equations of electron motion (Eqs. (27–31) presented in the “Methods” section).

**Figure 2:**
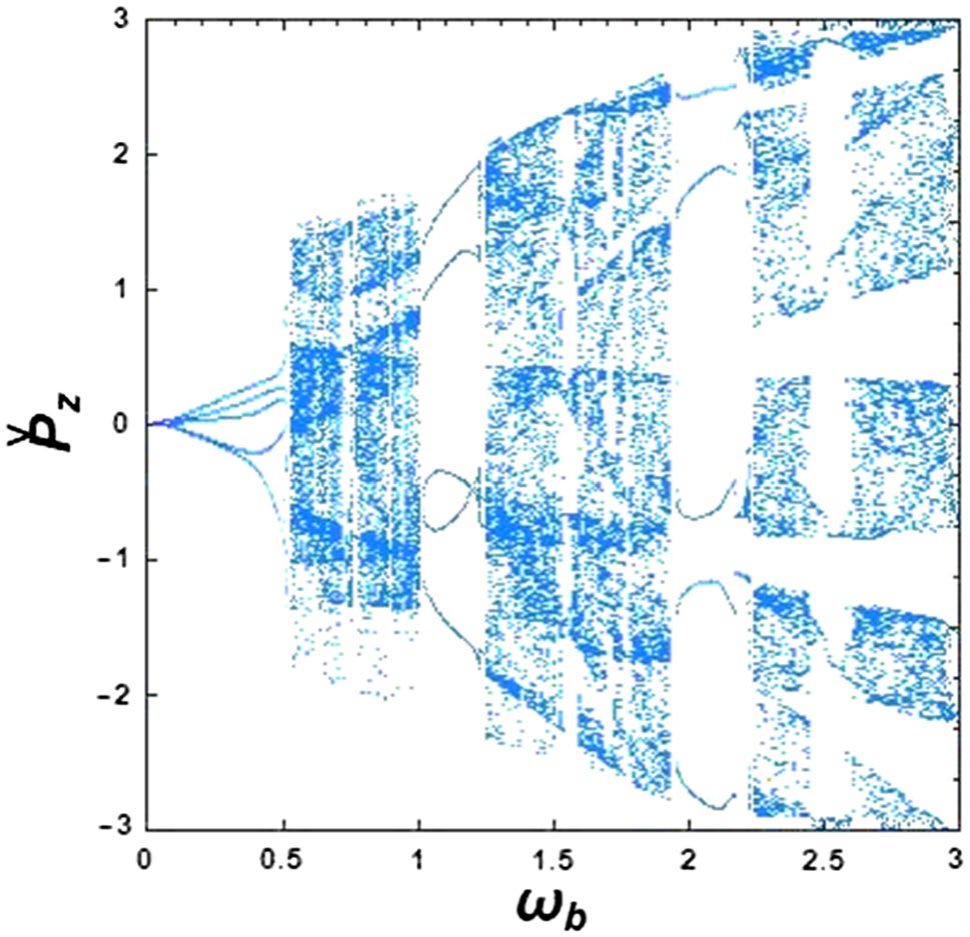
Simulated bifurcation diagram of the XFEL based on an optical undulator: the effect of normalized beam frequency on the stability of electron motions. The constant parameters are considered as ${\Omega }_{{\text{w}}} = 0.5$, ${\Omega }_{0} = 1.0$ and ${\upomega }_{{\text{i}}} = 1.4$.

To investigate the influence of the self-electric and self-magnetic fields on chaotic motion, a bifurcation diagram for the optical undulator in the presence of a magnetized ion-channel is presented (see Fig. 2). The normalized beam frequency, ${\upomega }_{{\text{b}}}$, has been considered as a control parameter in Fig. 2. Here, the phase plane includes both chaotic orbits and regular orbits. As can be seen in this figure, with the increase of the normalized beam frequency, ${\upomega }_{{\text{b}}} = 0.48$, the irregular and chaotic electron motion emerge, distinctively. The cascade of regular regimes turns into chaos at $0.48 < {\upomega }_{{\text{b}}} < 1.03$, and $1.22 < {\upomega }_{{\text{b}}} < 1.93$, as well as ${\upomega }_{{\text{b}}} > 2.23$ regions. The fact that there are chaotic trajectories, indicates that the system is non-integrable. This effect can be attributed to sideband instability, and in this case, the single-frequency output radiation changes to a broadband one, and chaos begins. However, it was found that it is possible to prevent chaos in an XFEL by changing the beam density (or beam frequency) in some specific regions.

**Figure 3:**
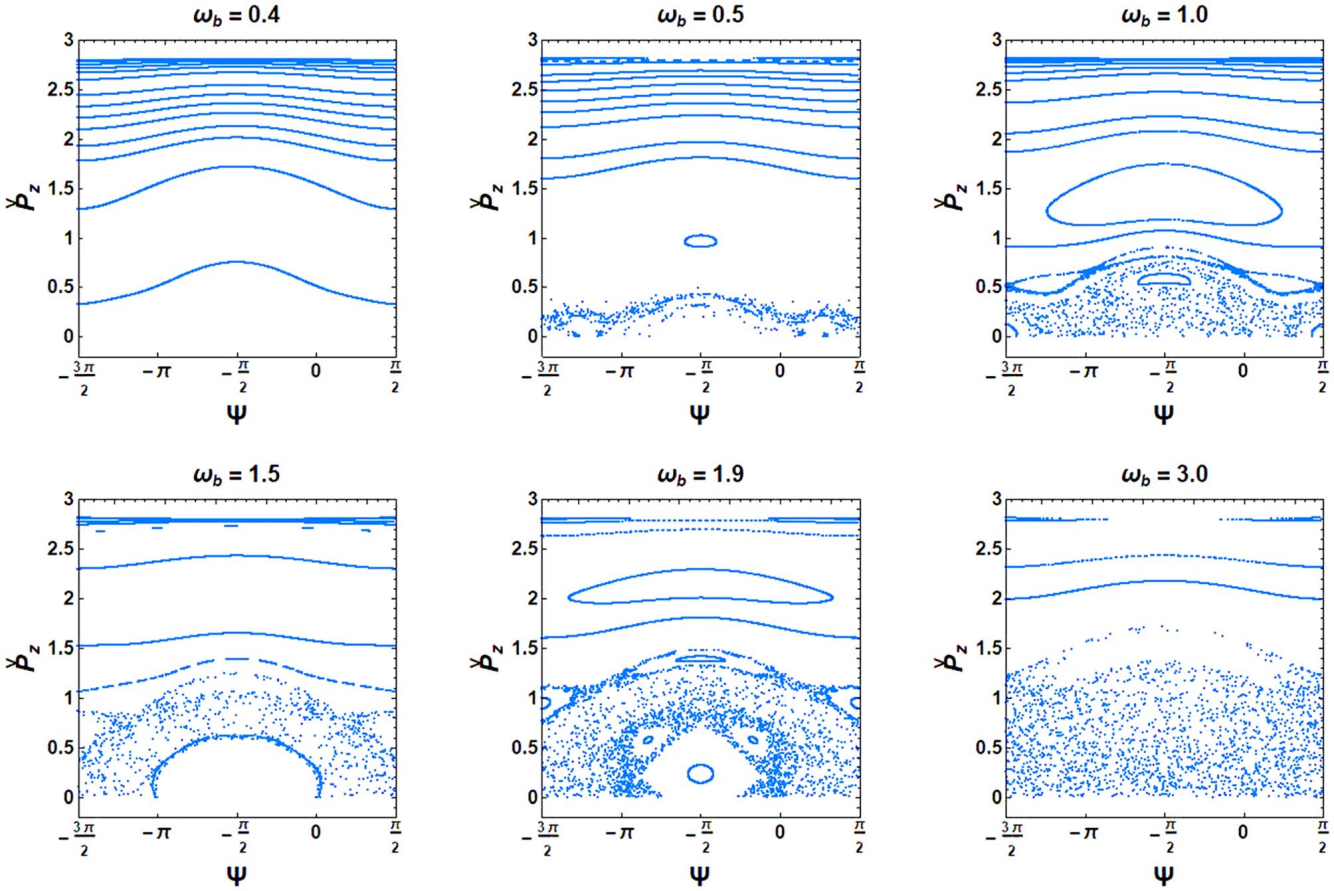
Poincaré cross-section plots in the phase plane $\left( {{\Psi },{{ \check{p}}}_{{\text{z}}} } \right)$ for an optical undulator in the XFEL. The constant parameters are considered as ${\Omega }_{{\text{w}}} = 0.5$, ${\Omega }_{0} = 1.0$, and ${\upomega }_{{\text{i}}} = 1.4$.

To confirm the behavior obtained by the bifurcation diagram, Poincaré maps were plotted. The Poincaré cross-section of the optical undulator with a magnetized ion-channel background for various beam frequency parameters is plotted in Fig. 3. In an XFEL with an optical undulator driven by a high-density e-beam, the electron motion can be significantly modified by the self-fields. Under these conditions, when the beam self-field effects increase, the electron motions become unstable, so the phase plane indicates chaotic behavior. As seen in Fig. 3, increasing the normalized beam frequency causes the regular trajectories to turn into chaos rapidly. In fact, a chaotic phase exists in the high beam density regime. Therefore, it is crucial to evaluate the influence of the magnetized ion-channel on the quenching of chaos in cases where the beam self-fields are dominant under conditions of high e-beam density.

**Figure 4:**
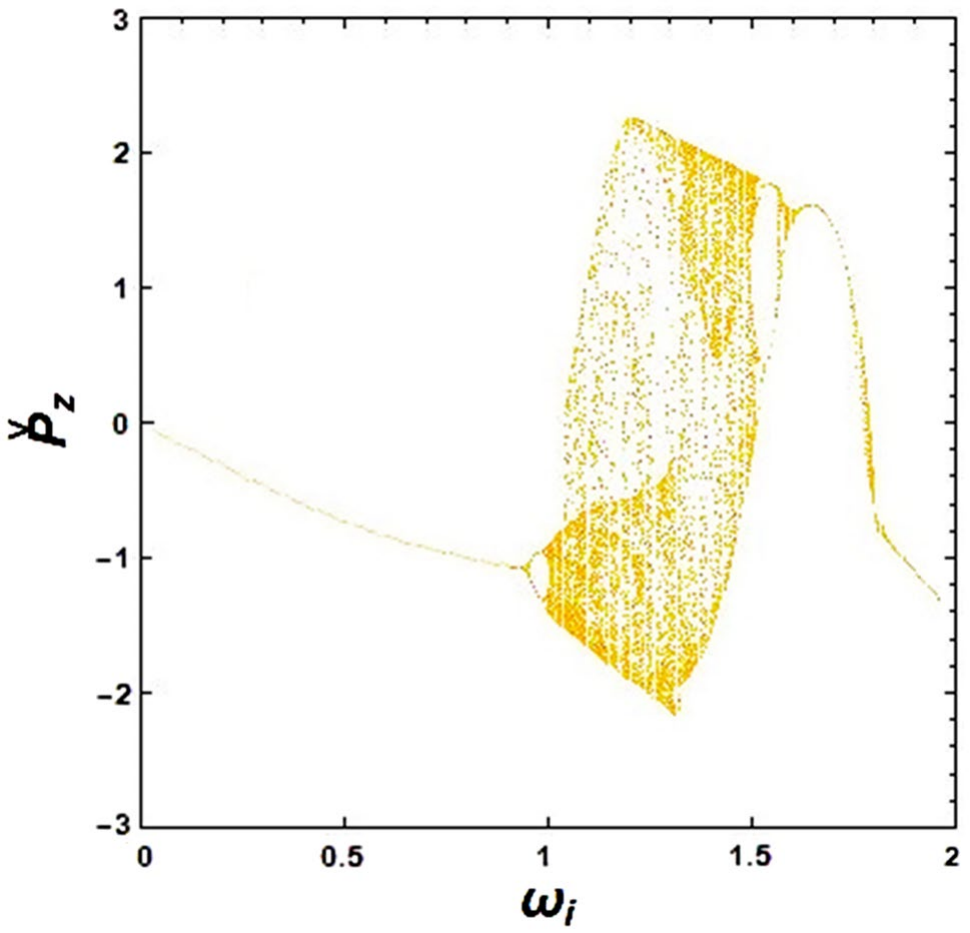
Simulated bifurcation diagram of the XFEL based on an optical undulator: the effect of normalized ion-channel frequency on the electron motions. The constant parameters are considered as ${\Omega }_{{\text{w}}} = 0.5$, ${\Omega }_{0} = 1.0$ and ${\upomega }_{{\text{b}}} = 0.5$.

To investigate the effect of the ion-channel background on the chaotic motion in the presence of an optical undulator, a bifurcation diagram has been drawn in Fig. 4. As can be seen in this figure, a cascade of periodic regimes turns into chaos for a specific range of ${\upomega }_{{\text{i}}}$ values before returning to periodicity again. Actually, there is only a chaotic window in $0.98 < {\upomega }_{{\text{i}}} < 1.46$ region, and the electron has a regular motion for other values of ${\upomega }_{{\text{i}}}$. This indicates that ion-channel frequency (or ion-channel density) has a remarkable effect on controlling the chaotic behavior of electrons in XFELs with optical undulators.

**Figure 5:**
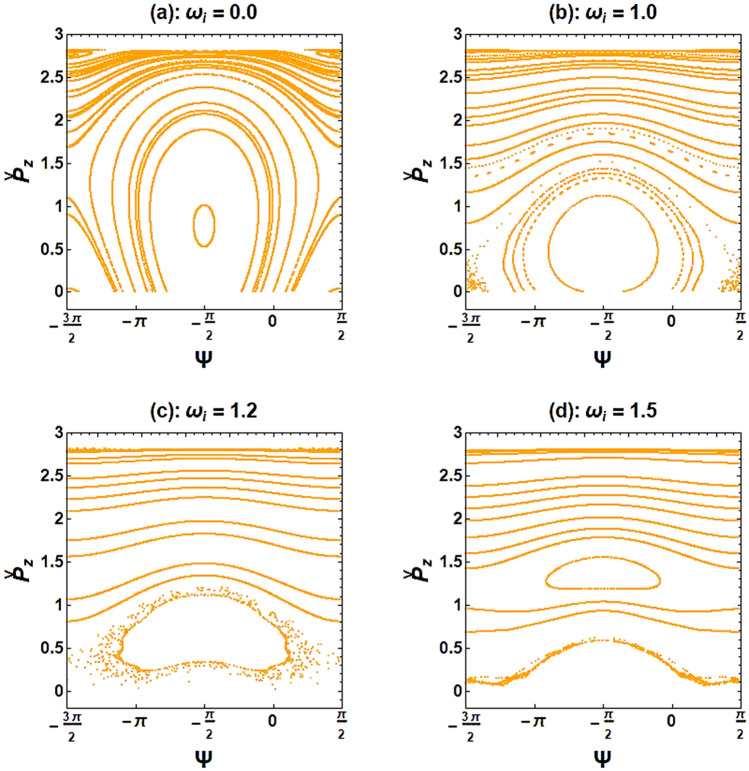
Poincaré cross-section plots of the XFEL based on an optical undulator for different normalized ion-channel frequencies. The selected parameters are the same as in Fig. 4.

To confirm the behavior obtained by the bifurcation diagram, Poincaré maps were drawn in Fig. 5. As shown in this figure, in the presence of the ion-channel background, the chaos regime appears at ${\upomega }_{{\text{i}}} = 1.0$. As the frequency of the ion-channel increases, the chaotic behavior of the system enhances until the electron trajectories reach the maximum instability at ${\upomega }_{{\text{i}}} = 1.2$, as shown in Fig. 5c. In fact, chaos appears in a part of the phase space, leading to divergent trajectories. In other words, the orderly part of the cross-section is strongly shrunk near the critical point of ion-channel frequency in the resonance region. Finally, at ${\upomega }_{{\text{i}}} = 1.5$, the electron returns to regular movements and the electron trajectories remain stable, as shown in Fig. 5d. These results are in agreement with the bifurcation diagram.

**Figure 6:**
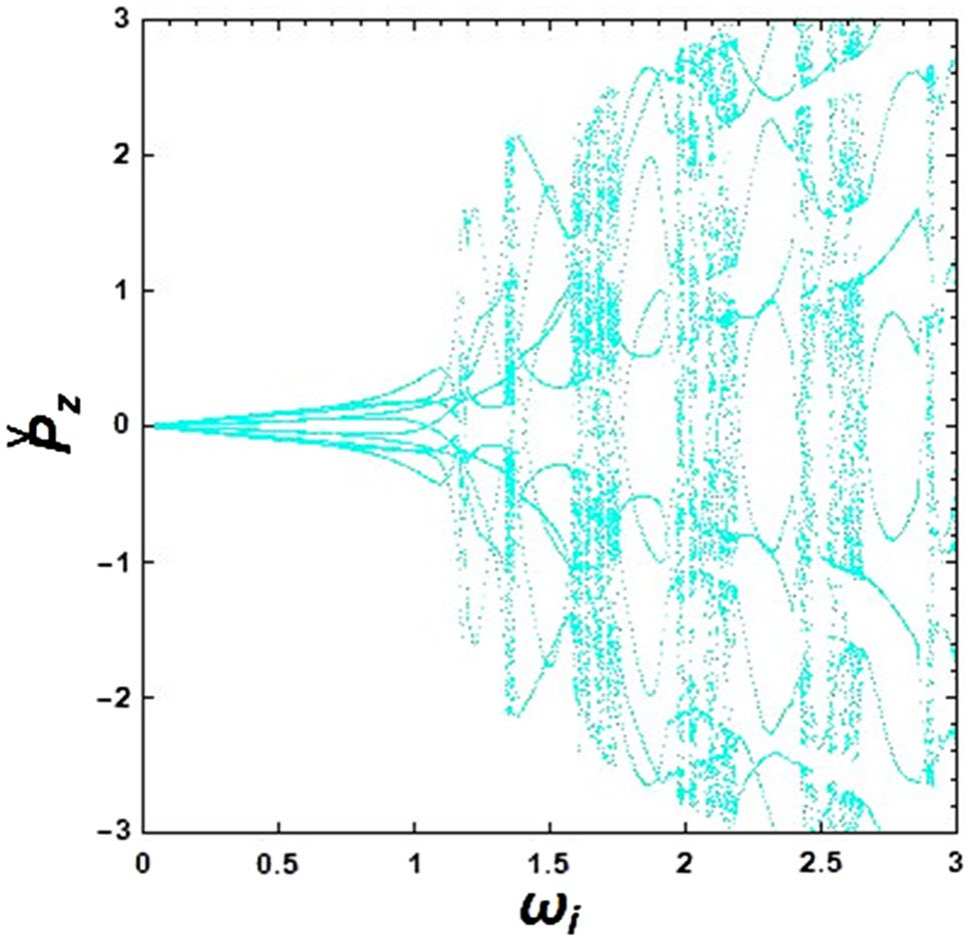
Simulated bifurcation diagram of the XFEL based on an optical undulator: the effect of normalized ion-channel frequency on high-energy electron motions (${\upgamma } = 8$). The constant parameters are considered as ${\upomega }_{{\text{b}}} = 0.5$, ${\Omega }_{0} = 1.0$ and ${\Omega }_{{\text{w}}} = 0.5$.

**Figure 7:**
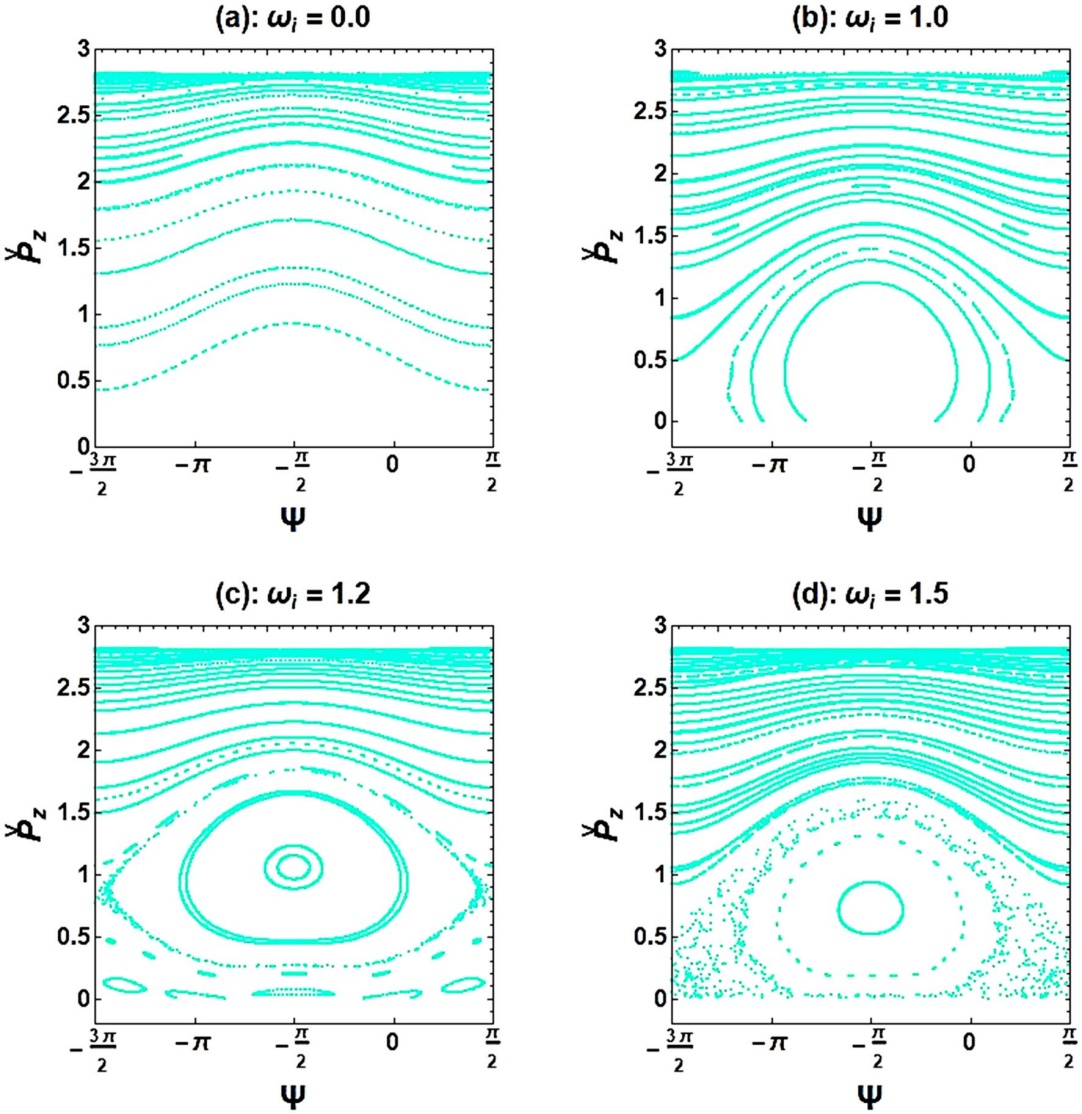
Poincaré cross-section plots of the XFEL based on an optical undulator with high-energy electron beam (${\upgamma } = 8$). The selected parameters are the same as in Fig. 6.

**Figure 8:**
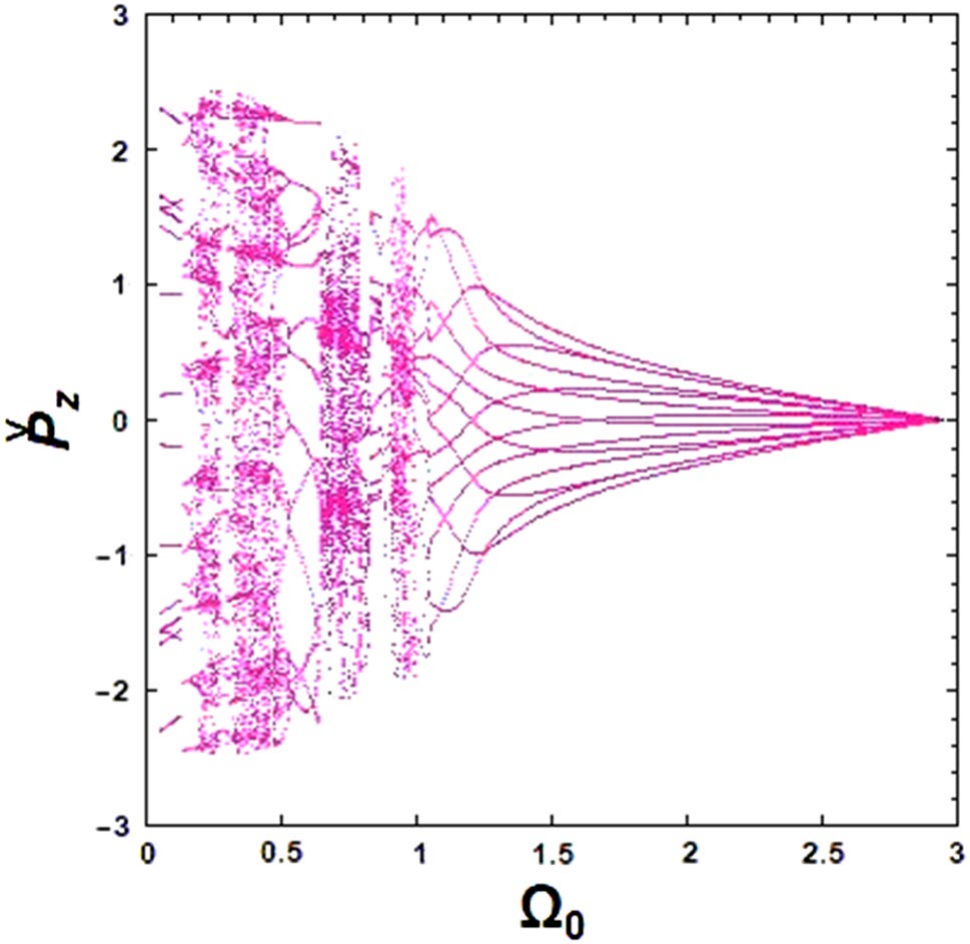
Simulated bifurcation diagram of the XFEL based on an optical undulator: the effect of normalized axial guide magnetic field frequency on the electron motion. The constant parameters are considered as ${\Omega }_{{\text{w}}} = 0.5$, $\omega_{b} = 0.5$ and ${\upomega }_{{\text{i}}} = 1.4$.

We were curious about the behavior of electrons with higher energy in such a structure. To achieve this case, we increased the electron energy from 3 (in Fig. 4) to 8 and re-ploted the bifurcation diagram for γ = 8. As can be seen in Fig. 6, the transition to chaos occurs at a larger critical point for the ion-channel frequency, *i.e*., ${\upomega }_{{\text{i}}} = 1.23$, and then by increasing ${\upomega }_{{\text{i}}}$, the electron motions become chaotic, which is contrary to what was found in Fig. 4. This indicates that changing the energy of the incident e-beam creates distinct chaos. Actually, depending on the initial conditions, the chaos window of the system becomes narrower or wider.

Poincaré maps are also drawn for better comparison of the ion-channel effect on the stability of electrons with different kinetic energies (*i.e*., γ = 3 and 8) (see Fig. 7). When there is no ion-channel background, Figs. 5a and 7a are in agreement to each other on the regularity and stability of electron trajectories for both low and high beam energy values. However, when comparing Fig. 5b and Fig. 7b, it can be found that for the same normalized ion-channel frequency (${\upomega }_{{\text{i}}} = 1.0$), the electron with higher kinetic energy has more regular trajectories. As can be seen in Fig. 7c, the electron motion does not enter to chaos regime until ${\upomega }_{{\text{i}}} = 1.2$, whereas in Fig. 5c, the electron motion experiences the highest amount of chaos at this critical point. Moreover, as the ion-channel frequency increases (Fig. 7d), the chaotic motions of energetic electrons grow rapidly, and the system’s instability increases.

In Fig. 8, another bifurcation diagram is presented for an optical undulator employing a magnetized ion-channel. The diagram considers the normalized guide magnetic field frequency as the control parameter. When there is no guide magnetic field (i.e., ${\Omega }_{0} = 0$), the dynamic behavior of the system is chaotic. This highlights the crucial role of the axial magnetic field in stabilizing the electron trajectories. By changing the frequency of the axial magnetic field, the system passes from a chaotic state to a completely periodic behavior at ${\Omega }_{0} = 1.12$. This type of behavior is desirable among dynamic behaviors due to its predictable nature.

**Figure 9:**
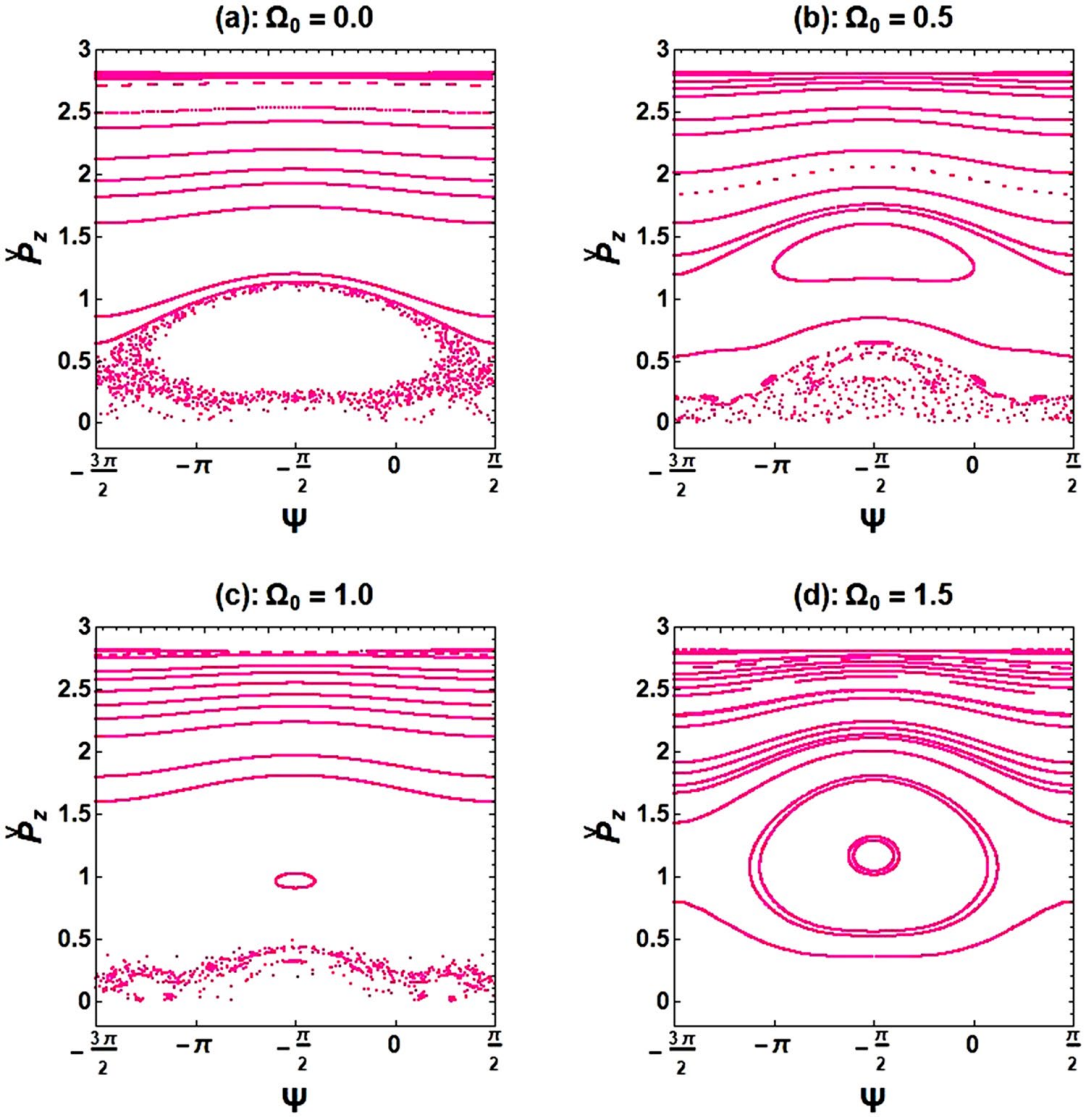
Poincaré cross-section plots for the XFEL based an optical undulator with a magnetized ion-channel in the presence of different normalized axial magnetic field frequencies. The constant parameters are the same as in Fig. 8.

To confirm the behavior observed in the bifurcation diagram, Poincaré maps were re-plotted in Fig. 9. These maps demonstrate the influence of the axial-guide magnetic field on chaotic states. As can be seen in Fig. 9a, even when there is no axial guide magnetic field, but with the presence of ion-channel background, there is still chaotic behavior in the electron motions. Further results revealed that an increase in the frequency of the guide magnetic field leads to a transition from non-periodic (unbounded) to periodic (closed) phase space (see Fig. 9b–d). Of course, it was previously shown in Figs. 4 and 5 that when the magnetized ion-channel is present, the system’s dynamics can be turned into regular and periodic behavior by increasing the frequency of the ion-channel.

**Figure 10:**
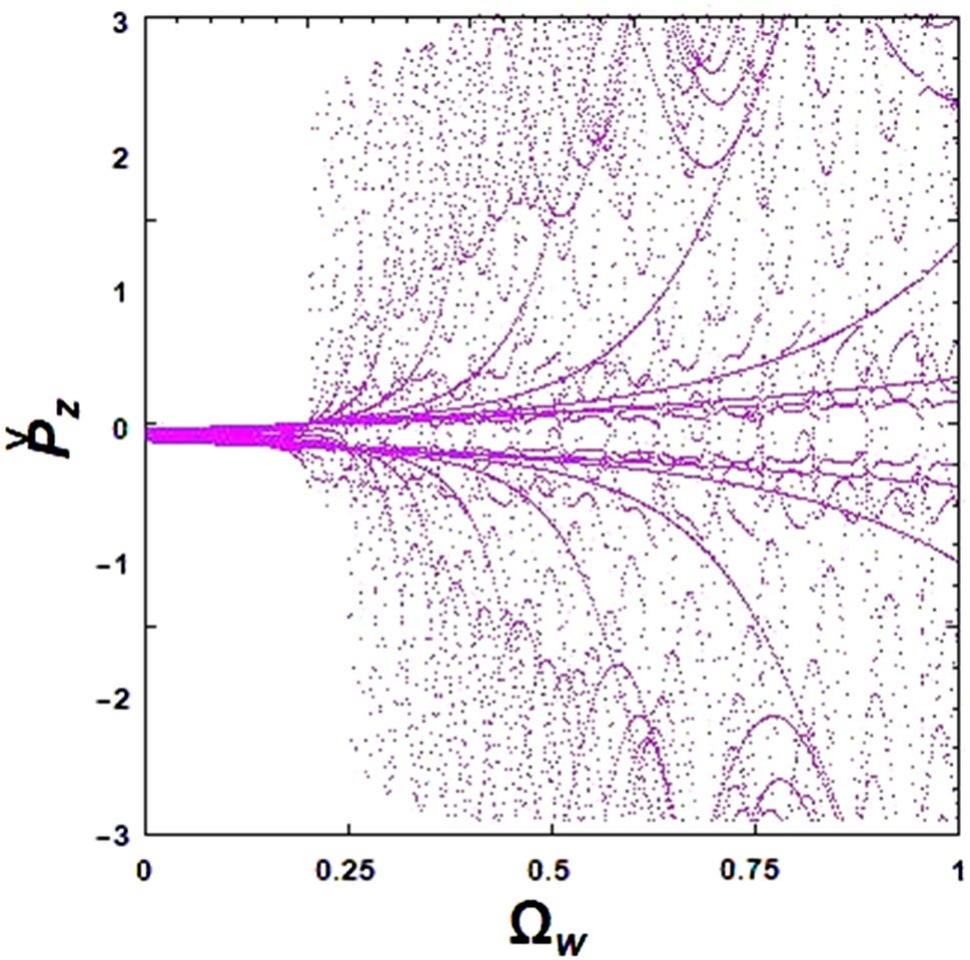
Simulated bifurcation diagram of the XFEL based an optical undulator: the effect of undulator field frequency on the electron motion. The constant parameters are considered as ${\Omega }_{0} = 1.0$, $\omega_{b} = 0.5$ and ${\upomega }_{{\text{i}}} = 1.4$.

The stability of electron trajectories is significantly influenced by the undulator field frequency, as depicted in the bifurcation diagram in Fig. 10. This figure illustrates that as the undulator field frequency increases, regular motion becomes highly unstable after several bifurcation sequences. A rapid expansion of the normalized axial momentum p̌_z_, is seen at the critical normalized undulator field frequency of ${\Omega }_{{\text{w}}} = 0.25$. The cascade of periodic regimes finally turns into chaos.

**Figure 11:**
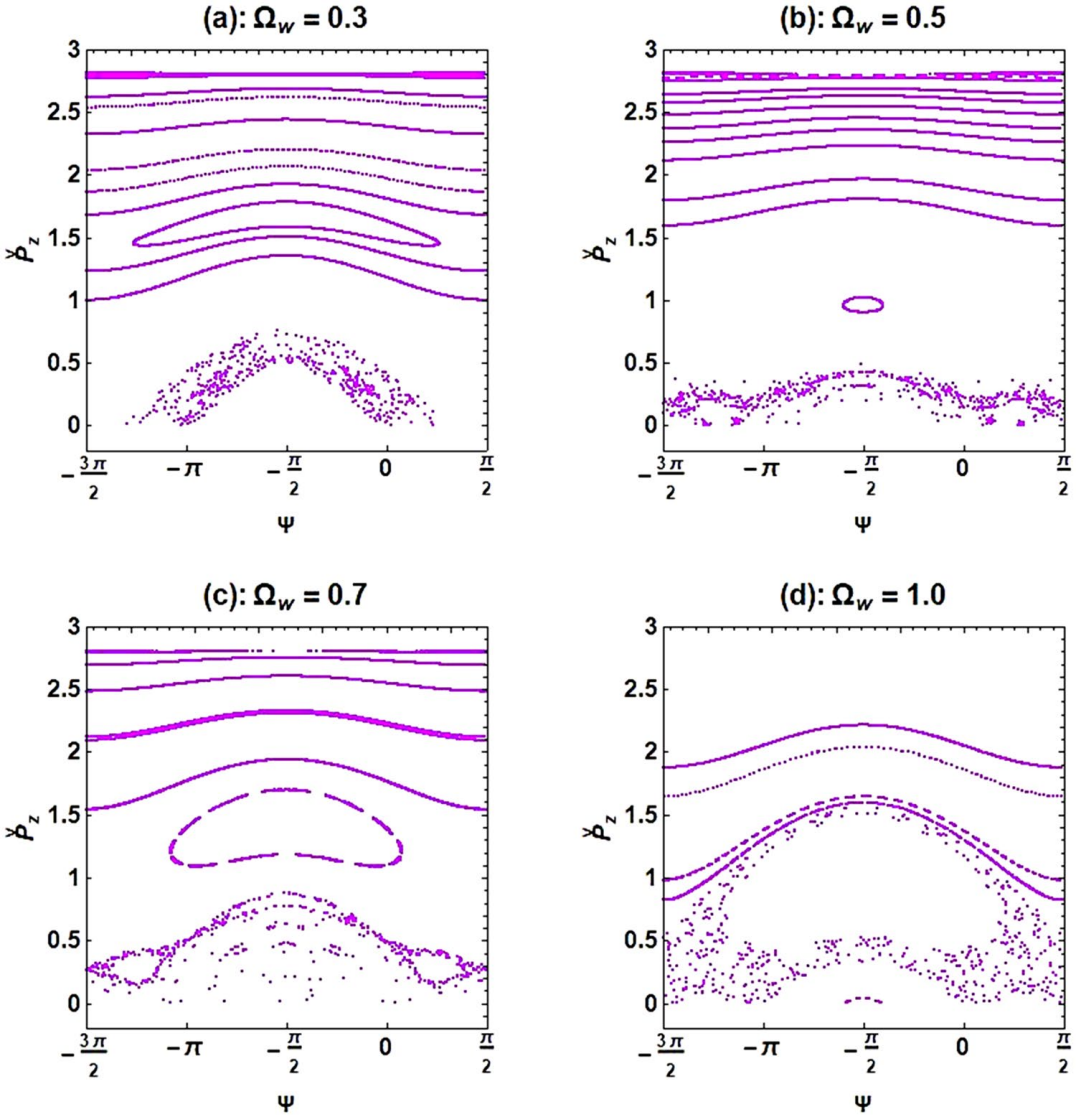
Poincaré cross-section plots for the XFEL based on an optical undulator with different undulator field frequencies. The constant parameters are the same as in Fig. 10.

Figure 11 displays the Poincaré cross-section of an optical undulator with different undulator field frequencies, ${\Omega }_{{\text{w}}}$, where both the axial magnetic field and the ion-channel background are used to guide the e-beam. As can be seen in this figure, with an increase in the undulator field frequency, the electron motions become more unstable. This happens because the stronger undulator field tends to deflect the electron motion from its path.

## Discussion

A comprehensive analysis has been conducted on the dynamics of the XFEL employing an optical undulator (with a linearly polarized laser pulse) and a magnetized ion-channel background. In our study, it can be expected that the XFEL operates in the classical regime where a large number of photons are emitted before the electron energy changes significantly. As is known, the quantum effects can be neglected if the photon momentum recoil is not larger than the beam momentum spread^44,45^. As demonstrated in Eqs. (33–37), the numerical values of parameters used in our scheme, which are in good agreement with the experimental values^44–46^, are sufficient to obtain a matching classical dynamic of the system. The Hamiltonian of the system was obtained for the test electron in the interaction region, and the chaotic behavior of the electron was examined through bifurcation diagrams and Poincaré maps. If the number of constants of motion is less than the freedom degree of the Hamiltonian, the equations become non-integrable, hence chaos appears in the system’s dynamics. The Pioncaré map is a valuable tool for analyzing non-integrable systems since the dimension of the Pioncaré cross-section is always one unit less than the phase space dimension of the interaction region. In the Poincaré cross-section plot, the evolution of chaos is revealed, and in the bifurcation diagram, chaos emerges with a rapid expansion of the attractor of the system at a critical parameter. It occurs simultaneously with the triggering of high-density beams in XFELs. In fact, chaos is identified through the directional exponential stretching of the trajectory of a non-linear dynamical system. Many XFEL experiments use an axial magnetic field to guide the e-beam axially when operating at high beam currents. Here, we have proposed a novel method (using a magnetized ion-channel background) to improve control over e-beam stability and optical undulator focusing, as well as prevent destructive chaotic conditions in the optical undulators.

Due to using a high current electron beam (or a high e-beam density) in a high-power radiation mechanism, we examined the self-field effects on the chaotic behavior of XFELs. Besides, the effect of the guidance system, including ion-channel background and axial magnetic field, on the stability of the electron motion was investigated. For these purposes, the bifurcation diagram and Poincaré maps were plotted. Our findings indicated that increasing the frequency of the e-beam causes a bifurcation transition from a stable to a chaotic state. This effect can be attributed to sideband instability in which the single-frequency output radiation shifts to a broadband one and chaos begins. In other words, when the beam self-fields are trivial (*i.e.,* in low current e-beam), electron trajectories are stable, but when the effects of self-induced fields increase (*i.e.,* in high current e-beam), causing electron motions to become unstable and chaotic. In fact, chaos arises by coupling the electron betatron oscillation to the undulator wave (input laser pulse). The results indicated that as e-beam current increases, this coupling becomes stronger. This reveals that it is possible to prevent chaos in XFELs by adjusting the e-beam density (or e-beam frequency). It had been deduced that the chaos depends only on the beam density value. However, the following results have revealed that to prevent chaos, the ion-channel density value (or ion-channel frequency) and the magnetic field strength (or axial magnetic field frequency) can be adjusted. This will ensure that the movement of the test electron in the system remains regular and non-chaotic, ultimately leading to the creation of an intense single-frequency X-ray pulse. To study the ion-channel effect, we examined the behavior of electrons with increasing ion-channel density (or ion-channel frequency) while maintaining a constant frequency of axial magnetic field. Our simulations indicated that when the normalized frequency of the ion-channel is $0.98 \le {\upomega }_{{\text{i}}} \le 1.46$, the electron motions become chaotic. However, for any other values of ${\upomega }_{{\text{i}}}$, all trajectories are regular with very small fluctuations. Moreover, when comparing the stability of motion between an electron with high energy (${\upgamma } = 8$) and an electron with low energy (${\upgamma } = 3$) in the XFEL, it was found that the electron with higher kinetic energy has more regular trajectories when ${\upomega }_{{\text{i}}} = 1.0$. As the frequency of the ion-channel increases, the chaotic trajectories of the energetic electron grow rapidly, while the electron with low kinetic energy maintains its regular motion.

If the frequency of the guide magnetic field increases while keeping the frequency of ion-channel constant, ${\upomega }_{{\text{i}}} = 1.4$, the chaotic trajectories decrease for a normalized axial magnetic field ${\Omega }_{0} > {\Omega }_{0}^{cr}$ (${\Omega }_{0}^{cr}$ refers to a normalized axial magnetic field where the disordered paths transform to ordered paths). The exact value of ${\Omega }_{0}^{cr}$ can be obtained from the bifurcation diagram, and it was found to be 1.12 for the selected initial parameters. Increasing the frequency of the ion-channel or the frequency of the guide magnetic field in the resonance region can cause the chaos to disappear. At sufficient frequencies of the axial magnetic field and ion-channel, electron motion becomes completely stable. Further results indicate that using an axial magnetic field, ${\Omega }_{0} = 1.0$, and the ion-channel, ${\upomega }_{{\text{i}}} = 1.4$, simultaneously for the case of ${\upomega }_{{\text{b}}} = 0.5$ can lead to chaotic behavior. As an important result, in weak or medium regimes of beam current intensity, omitting the guide magnetic field still results in chaos in electron motions. However, by omitting the ion-channel guiding, electron trajectories become nonchaotic and completely regular. Further investigations indicated that stronger undulator fields lead to more unstable electron motions. The chaotic motion of an electron propagating through the optical undulator with a weak undulator magnetic field is caused only by the diamagnetic and paramagnetic effects of the undulator self-induced magnetic field^15^. Therefore, one would expect to see a more chaotic behavior of the electron in a stronger undulator field, which can lead to a stronger self-magnetic field.

## Methods

Figure 1 shows a schematic illustration of the proposed scheme. In the desired configuration, an intense relativistic e-beam (propagating with ${\mathbf{v}} = v_{\parallel } \hat{e}_{z}$) is propagated through a linearly polarized laser pulse (as an optical undulator) described by^15,38^

1 $$ {\mathbf{E}}_{w} = - \frac{{\omega_{w} }}{{ck_{w} }}B_{w} \sin \left( {k_{w} z + \omega_{w} t} \right) \hat{e}_{x} , $$

2 $$ {\mathbf{B}}_{w} = B_{w} \sin \left( {k_{w} z + \omega_{w} t} \right) \hat{e}_{y} , $$

where $B_{w}$ denotes the amplitude of the undulator magnetic field, and $\left( {\omega_{w} , k_{w} } \right)$ describes the angular frequency and wave vector of the undulator, respectively. Due to operation in the regime of high electron beam density, the effects of self-fields on the XFEL performance become more significant. To model self-fields, we assume the e-beam density to be constant, $n_{b} \left( r \right) = {\text{const}},$ where $r_{b}$ is the beam radius. The beam self-electric and self-magnetic fields

3 $$ {\mathbf{E}}_{s} = - 2\pi en_{b} \left( {x \hat{e}_{x} + y \hat{e}_{y} } \right), $$

4 $$ {\mathbf{B}}_{s} = 2\pi en_{b} \beta_{\parallel } \left( {y \hat{e}_{x} - x \hat{e}_{y} } \right), $$

resulting from the uniform density are obtained from solving Maxwell’s equations: $\nabla .{\mathbf{E}}_{s} = - 4\pi en_{b} \left( r \right)$ and $\nabla \times {\mathbf{B}}_{s} = 4\pi {\mathbf{J}}/c$, where ${\mathbf{J}}\left( { = - en_{b} c\beta_{\parallel } \hat{e}_{z} } \right)$ is the electron current density, and $\beta_{\parallel } \left( { = v_{\parallel } /c} \right)$ is the normalized electron axial velocity. A beam guidance system consisting of a uniform axial magnetic field, ${\mathbf{B}}_{0} = B_{0} \hat{e}_{z}$, and an electrostatic ion-channel field with uniform density $n_{i}$ and ion charge + *e*,

5 $$ {\mathbf{E}}_{i} = + 2\pi en_{i} \left( {x \hat{e}_{x} + y \hat{e}_{y} } \right), $$

is also employed to collimate the relativistic e-beam and focus the electrons against the self-repulsive electrostatic force generated by the self-fields.

The Hamiltonian of a test relativistic electron in this configuration can be represented as,

6 $$ H = \left[ {\left( {c{\mathbf{P}} + e{\mathbf{A}}} \right)^{2} + m^{2} c^{4} } \right]^{1/2} - e\left( {\phi_{i} + \phi_{s} } \right) \equiv \gamma mc^{2} - e\left( {\phi_{i} + \phi_{s} } \right), $$

where ${\mathbf{P}}$ and $p\left( { = {\mathbf{P}} + e{\mathbf{A}}/c} \right)$ are the canonical and mechanical momentum, respectively, and $\gamma = \left( {1 + (p/mc} \right)^{2} )^{1/2}$ is the Lorentz parameter. ${\mathbf{A}}\left( { = {\mathbf{A}}_{w} + {\mathbf{A}}_{0} + {\mathbf{A}}_{s} } \right)$ is the combination of the vector potential of the laser undulator, the vector potential of the guide magnetic field, and the vector potential of the self-magnetic field as follows,

7 $$ {\mathbf{A}}_{w} = {\mathbf{A}}_{0w} \cos \left( {k_{w} z + \omega_{w} t} \right) \hat{e}_{x} , $$

8 $$ {\mathbf{A}}_{0} = B_{0} x \hat{e}_{y} , $$

9 $$ {\mathbf{A}}_{{\varvec{s}}} = \beta_{\parallel } \phi_{s} \hat{e}_{z} , $$

where ${\mathbf{A}}_{0w} = - B_{w} /k_{w}$ is the amplitude of the vector potential associated with the laser undulator, and

10 $$ \phi_{s} = \frac{{mk_{w}^{2} c^{2} \omega_{b}^{2} }}{4e}\left( {x^{2} + y^{2} } \right) $$

is the electrostatic potential caused by the e-beam, and $\omega_{b} \left( { \equiv \left( {4\pi e^{2} n_{b} /mk_{w}^{2} c^{2} } \right)^{1/2} } \right)$ is the normalized beam frequency. In addition,

11 $$ \phi_{i} = - \frac{{mk_{w}^{2} c^{2} \omega_{i}^{2} }}{4e}\left( {x^{2} + y^{2} } \right) $$

is the electrostatic potential caused by the ion-channel background, where $\omega_{i} \left( { \equiv \left( {4\pi e^{2} n_{i} /mk_{w}^{2} c^{2} } \right)^{1/2} } \right)$ is the normalized ion-channel frequency. For notational convenience, we need to provide other normalized variables and parameters: $\overline{P}_{x,y,z} = P_{x,yz} /mc$**,** $\left( {\overline{x}, \overline{y}, \overline{z}} \right) = k_{w} \left( {x, y, z} \right)$, $\tau = ck_{w} t$, $\Omega_{0,w} = eB_{0,w} /mk_{w} c^{2}$, and $\overline{\phi }_{s,i} = e\phi_{s,i} /mc^{2}$**.** Therefore, the normalized Hamiltonian $\overline{H}\left( { = H/mc^{2} } \right)$ is expressed as follows,

12 $$ \overline{H} = \left\{ {\left[ {\overline{P}_{x} - \Omega_{w} \cos \left( {\overline{z} + \beta_{p} \tau } \right)} \right]^{2} + \left[ {\overline{P}_{y} + \Omega_{0} \overline{x}} \right]^{2} + \left[ {\overline{P}_{z} + \overline{\phi }_{s} \beta_{\parallel } } \right]^{2} + 1} \right\}^{1/2} - \left( {\overline{\phi }_{i} + \overline{\phi }_{s} } \right), $$

where $\beta_{p} ( \equiv \omega_{w} /ck_{w} )$ shows the normalized phase velocity of the laser undulator. Canonical transformations help to specify resonance conditions by finding constants of motion^47^. The transformation with the introduced variables $\left( {\alpha , \beta , \overline{z}^{\prime } , \overline{P}_{\alpha } , \overline{P}_{\beta } , \overline{P}_{{\overline{z}{\prime} }} } \right)$ is as follows,

13 $$ \overline{x} = \left( {\frac{{2\overline{P}_{\alpha } }}{{\Omega_{0} }}} \right)^{1/2} \sin \left( {\alpha + \overline{z}^{\prime}} \right) - \left( {\frac{{2\overline{P}_{\beta } }}{{\Omega_{0} }}} \right)^{1/2} \cos \left( {\beta - \overline{z}^{\prime}} \right), $$

14 $$ \overline{y} = \left( {\frac{{2\overline{P}_{\beta } }}{{\Omega_{0} }}} \right)^{1/2} \sin \left( {\beta - \overline{z}^{\prime}} \right) - \left( {\frac{{2\overline{P}_{\alpha } }}{{\Omega_{0} }}} \right)^{1/2} \cos \left( {\alpha + \overline{z}^{\prime}} \right), $$

15 $$ \overline{z} = \overline{z}^{\prime}, $$

16 $$ \overline{P}_{x} = \left( {2\Omega_{0} \overline{P}_{\alpha } } \right)^{1/2} \cos \left( {\alpha + \overline{z}^{\prime}} \right), $$

17 $$ \overline{P}_{y} = \left( {2\Omega_{0} \overline{P}_{\beta } } \right)^{1/2} \cos \left( {\beta - \overline{z}^{\prime}} \right), $$

18 $$ \overline{P}_{z} = \overline{P}_{{\overline{z}{\prime} }} + \overline{P}_{\alpha } - \overline{P}_{\beta } . $$

Then, the new Hamiltonian is given by,

19 $$ \overline{H} = \left[ {2\Omega_{0} \overline{P}_{\alpha } + \Omega_{w}^{2} /2 - \Omega_{w} \left( {2\Omega_{0} \overline{P}_{\alpha } } \right)^{1/2} \cos \left( {\alpha - \beta_{p} \tau } \right) + \left( {\overline{P}_{{\overline{z}{\prime} }} + \overline{P}_{\alpha } - \overline{P}_{\beta } + \overline{\phi }_{s} \beta_{\parallel } } \right)^{2} + 1} \right]^{1/2} - \left( {\overline{\phi }_{i} + \overline{\phi }_{s} } \right), $$

where the new potentials, $\overline{\phi }_{i}$ and $\overline{\phi }_{s}$**,** are represented by substituting Eqs. (13–18) into Eqs. (10) and (11) as follows,

20 $$ \overline{\phi }_{i} = - 2\varepsilon_{i} \Omega_{0} \left[ {\overline{P}_{\alpha } + \overline{P}_{\beta } - 2\left( {\overline{P}_{\alpha } \overline{P}_{\beta } } \right)^{1/2} \sin \left( {\alpha + \beta } \right)} \right], $$

21 $$ \overline{\phi }_{s} = 2\varepsilon_{b} \Omega_{0} \left[ {\overline{P}_{\alpha } + \overline{P}_{\beta } - 2\left( {\overline{P}_{\alpha } \overline{P}_{\beta } } \right)^{1/2} \sin \left( {\alpha + \beta } \right)} \right]. $$

Moreover, $\varepsilon_{i,b} = \omega_{i,b}^{2} /4\Omega_{0}^{2}$ denotes the normalized parameters of the magnetized ion-channel and beam self-field strength. The Hamiltonian obtained in Eq. (19) is time-dependent, therefore, we need to remove the time variable through the appropriate canonical transformation, by representing the following function as^48^:

22 $$ F = \left( {\alpha - \beta_{p} \tau } \right)P_{\Phi } + \left( {\beta + \beta_{p} \tau } \right)P_{\Psi } + \overline{z}^{\prime}P_{{\overline{\zeta }}} , $$

where the new equations are rewritten as,

23 $$ P_{\Phi } = \overline{P}_{\alpha } , P_{\Psi } = \overline{P}_{\beta } , P_{{\overline{\zeta }}} = \overline{P}_{{\overline{z}{\prime} }} , \Phi = \alpha - \beta_{p} \tau , \Psi = \beta + \beta_{p} \tau , \overline{\zeta } = \overline{z}^{\prime}. $$

Substituting the above variables in Eq. (22), the transformed Hamiltonian $\overline{H}^{\prime}\left( { = \overline{H} + \partial F/\partial \tau } \right)$ can be written as follows,

24 $$ \overline{H}{\prime} = \left[ {2\Omega_{0} P_{\Phi } + \Omega_{w}^{2} /2 - \Omega_{w} \left( {2\Omega_{0} P_{\Phi } } \right)^{1/2} \cos \Phi + \left( {P_{{\overline{\zeta }}} + P_{\Phi } - P_{\Psi } + \overline{\phi }_{s} \beta_{\parallel } } \right)^{2} + 1} \right]^{1/2} - \left( {\overline{\phi }_{i} + \overline{\phi }_{s} } \right) - \beta_{p} \left( {P_{\Phi } - P_{\Psi } } \right), $$

where the transformed potentials, $\overline{\phi }_{i}$ and $\overline{\phi }_{s}$, are as follows,

25 $$ \overline{\phi }_{i} = - 2\varepsilon_{i} \Omega_{0} \left[ {P_{\Phi } + P_{\Psi } - 2\left( {P_{\Phi } P_{\Psi } } \right)^{\frac{1}{2}} \sin \left( {\Phi + \Psi } \right)} \right], $$

26 $$ \overline{\phi }_{s} = 2\varepsilon_{b} \Omega_{0} \left[ {P_{\Phi } + P_{\Psi } - 2\left( {P_{\Phi } P_{\Psi } } \right)^{\frac{1}{2}} \sin \left( {\Phi + \Psi } \right)} \right]. $$

According to Eq. (24), the Hamiltonian can be considered as the constant of motion corresponding to the entire energy conservation of the relativistic electron since it no longer depends on time. Moreover, it can be seen that the Hamiltonian does not depend on $\overline{\zeta }$ either, which means that $P_{{\overline{\zeta }}}$ is also one of the constants of motion. Therefore, the equations of motion for an electron propagating through the laser undulator using a magnetized ion-channel can be derived from Eqs. (24)–(26), as

27 $$ \frac{d\Phi }{{d\tau }} = \frac{{\check{p}_{z} }}{\gamma } + \frac{{\Omega_{0} }}{\gamma }\left( {1 - \frac{{\Omega_{w} }}{{2\left( {2\Omega_{0} P_{\Phi } } \right)^{1/2} }}{\text{cos}}\Phi } \right) + 2\Omega_{0} \left( {\frac{{\check{p}_{z} \beta_{\parallel } \varepsilon_{b} }}{\gamma } + \varepsilon_{i} - \varepsilon_{b} } \right)\left( {1 - \left( {\frac{{P_{\Psi } }}{{P_{\Phi } }}} \right)^{1/2} \sin \left( {\Phi + \Psi } \right)} \right) - \beta_{p} , $$

28 $$ \frac{d\Psi }{{d\tau }} = - \frac{{\check{p}_{z} }}{\gamma } + 2\Omega_{0} \left( {\frac{{\check{p}_{z} \beta_{\parallel } \varepsilon_{b} }}{\gamma } + \varepsilon_{i} - \varepsilon_{b} } \right)\left( {1 - \left( {\frac{{P_{\Phi } }}{{P_{\Psi } }}} \right)^{1/2} \sin \left( {\Phi + \Psi } \right)} \right) + \beta_{p} , $$

29 $$ \frac{{d\overline{\zeta }}}{d\tau } = \frac{{\check{p}_{z} }}{\gamma }, $$

30 $$ \frac{{dP_{\Phi } }}{d\tau } = - \frac{{\Omega_{w} }}{2\gamma }\left( {2\Omega_{0} P_{\Phi } } \right)^{1/2} \sin \Phi + 4\Omega_{0} \left( {P_{\Phi } P_{\Psi } } \right)^{1/2} \left( {\frac{{\check{p}_{z} \beta_{\parallel } \varepsilon_{b} }}{\gamma } + \varepsilon_{i} - \varepsilon_{b} } \right)\cos \left( {\Phi + \Psi } \right), $$

31 $$ \frac{{dP_{\Psi } }}{d\tau } = 4\Omega_{0} \left( {P_{\Phi } P_{\Psi } } \right)^{1/2} \left( {\frac{{\check{p}_{z} \beta_{\parallel } \varepsilon_{b} }}{\gamma } + \varepsilon_{i} - \varepsilon_{b} } \right)\cos \left( {\Phi + \Psi } \right), $$

and the normalized kinetic energy

32 $$ \gamma = \left[ {2\Omega_{0} P_{\Phi } + \frac{1}{2}\Omega_{w}^{2} + \Omega_{w} \left( {2\Omega_{0} P_{\Phi } } \right)^{\frac{1}{2}} \cos \Phi + \check{p}_{z}^{2} + 1} \right]^{1/2} , $$

where the normalized axial mechanical momentum $\check{p}_{z} \left( { = P_{{\overline{\zeta }}} + P_{\Phi } - P_{\Psi } + \overline{\phi }_{s} \beta_{\parallel } } \right)$ is provided to simplify the equations. We make bifurcation diagrams and Poincaré maps from Eqs. (27)–(31) to demonstrate the stability and chaos of electron motions in the laser undulator using the magnetized ion-channel background. The numerical error of ${\overline{\text{H}}}{\prime}$ is estimated and found to be less than $10^{ - 8}$ for each path. Therefore, ${\overline{\text{H}}}{\prime}$ can be considered as a constant of motion and its numerical value is taken 3. The interaction region has a 3D phase-space (${\Phi },{\Psi },{\text{ P}}_{{\Phi }}$). We have chosen the phase plan (${\Psi },{ }\check{p}_{{\text{z}}}$) with ${\text{P}}_{{\Phi }} = 0$ as the cross-section of Poincaré maps.

The quantum dynamics of a FEL is determined by a ‘‘quantum FEL parameter’’, $\overline{\rho }$, defined in terms of the classical parameter, $\rho$, as follows^49–51^

33 $$ \overline{\rho } = \rho \frac{\gamma mc}{{\hbar k_{w} }}, $$

which represents the ratio between the classical momentum spread and the one-photon recoil momentum. By introducing the Compton wavelength ($\lambda_{C} = 2\pi \hbar /mc = 0.024$ Å)^50^, we can rephrase Eq. (33) as follows,

34 $$ \overline{\rho } = \rho \frac{{\gamma \lambda_{w} }}{{\lambda_{C} }}, $$

where $\lambda_{w}$ is the wavelength of the laser pulse undulator. As far as $\overline{\rho } \ge 1$, quantum effects are negligible, and the system can be described through classical FEL-like equations^49,51^. For an XFEL based on a laser undulator, $\rho$ is estimated by^51^

35 $$ \rho = \frac{1}{\gamma }\left( {\frac{{\overline{\omega }_{b}^{2} a_{0w}^{2} }}{{16\omega_{w}^{2} }}} \right)^{\frac{1}{3}} , $$

where $\overline{\omega }_{b} \left( { \equiv \left( {4\pi e^{2} n_{b} /m} \right)^{1/2} } \right)$ is the average normalized beam frequency, and $a_{0w} = eA_{0W} /mc^{2}$ is the normalized amplitude of the laser undulator. By substituting the normalized beam frequency, $\omega_{b}$, the normalized magnetic field frequency, $\Omega_{w}$, and $\omega_{w} = ck_{w}$, Eq. (35) can be rewritten as

36 $$ \rho = \frac{1}{\gamma }\left( {\frac{{\omega_{b}^{2} \Omega_{w}^{2} }}{16}} \right)^{\frac{1}{3}} . $$

Substituting the Eq. (36) into Eq. (34) results in,

37 $$ \overline{\rho } = \frac{{\lambda_{w} }}{{\lambda_{C} }}\left( {\frac{{\omega_{b} \Omega_{w} }}{4}} \right)^{\frac{2}{3}} . $$

Considering $\lambda_{w} = 1\;{\upmu{\text{m}}}$ (for a laser undulator) and other numerical values of parameters presented in our scheme (${\upomega }_{{\text{b}}} = 0.5$, and ${\Omega }_{{\text{w}}} = 0.5$) leads to a $\overline{\rho }$ more than 1, which recovers the classical limit and guarantees that the quantum effects are negligible in this study. In this limit, the classical model of chaotic dynamics (Eqs. (27)–(31)) is expected to be valid.

## Conclusion

In summary, this study presented the chaotic motions of relativistic electrons in an X-ray free-electron laser using a linearly polarized laser undulator (as an optical undulator) in the presence of a magnetized ion-channel background. The interaction between the laser undulator and the intense relativistic electron beam is a highly non-linear phenomenon that can lead to chaotic dynamics. An appropriate method to investigate the non-linear dynamics in XFELs is to generate bifurcation diagrams and Poincaré maps. The Hamiltonian of the system was obtained analytically for the test electron in the interaction region, and the chaotic behavior of the electron was analyzed using bifurcation diagrams and Poincaré maps. The simulation results revealed that the intensity of the perturbation route from regular behavior to chaotic behavior depends on the beam density, axial magnetic field strength, ion-channel density, and pump laser undulator. It was found that when the electron beam self-fields are trivial, the electron trajectories are stable, but as the effects of the self-induced fields increase, the electron motions become unstable and chaotic. Further results indicated that in the presence of a dense e-beam in XFELs, the ion-channel density (or ion-channel frequency) and magnetic field strength (or magnetic field frequency) can adjust to prevent chaos in the system so that the movement of the test electron in the system is regular and non-chaotic. The proposed design can improve the performance of XFELs, which have potential applications in basic sciences, medicine, and industry.

## Data Availability

The data that supports the findings of this study are available within the article.
